# Non-human primate: the new frontier model of female reproductive engineering

**DOI:** 10.3389/fbioe.2025.1536750

**Published:** 2025-03-28

**Authors:** Yoon Young Kim, Jina Kwak, Byeong-Cheol Kang, Seung-Yup Ku

**Affiliations:** ^1^ Department of Obstetrics and Gynecology, Seoul National University Hospital, Seoul, Republic of Korea; ^2^ Institute of Reproductive Medicine and Population, Medical Research Center, Seoul National University, Seoul, Republic of Korea; ^3^ Department of Experimental Animal Research, Biomedical Research Institute, Seoul National University Hospital, Seoul, Republic of Korea; ^4^ Department of Translational Medicine, Seoul, Republic of Korea; ^5^ Department of Obstetrics and Gynecology, Seoul National University College of Medicine, Seoul, Republic of Korea

**Keywords:** non-human primate, reproductive engineering, reproduction, ovary, uterus

## Abstract

Reproductive engineering encompasses a range of advanced tissue engineering techniques aimed at addressing infertility that is non-curable with current assisted reproductive technology (ART). The use of animal models has been crucial for these advancements, with a notable preference for non-human primates (NHPs) given their genetic, anatomical, and physiological similarities to humans. Therefore, NHPs are invaluable for studying reproductive engineering. Thus, in reproductive studies, NHPs bridge the anatomical and physiological gaps between rodent models and humans. Their shared features with humans, such as menstrual cycles, placentation, and hormonal regulation, allow for more accurate modeling of reproductive physiology and pathology. These traits make NHPs indispensable in the exploration of reproductive engineering, including infertility treatments, genetic engineering, and uterine transplantation. Reproductive engineering is a transformative field that addresses infertility and enhances reproductive health. By leveraging the unique traits of NHPs, researchers can deepen their understanding of reproductive processes and refine ART techniques for human use. Advances in genetic engineering have enabled the creation of transgenic NHP models, which have been used to modify genes to investigate roles for various purposes, and the process, as mentioned earlier, is closely related to the ART technique, including fertility, embryogenesis, and pregnancy. Therefore, the relation to reproductive studies and the necessity of the NHP model are prerequisites for reproductive engineering. The engineering of NHPs is critically related to integrating ethical practices and exploring complementary methodologies. This review overviews the types of NHP frequently used and studies using NHP for reproductive engineering. These studies may suggest a broader way to use NHP for reproductive engineering.

## 1 Introduction

Despite significant advancements in assisted reproductive technologies (ARTs), often exemplified by *in vitro* fertilization (IVF), infertility rates have progressively increased among women. The causes of infertility vary and include ovarian aging, increased human longevity, survival after chemotherapy, delayed marriage, and conditions affecting younger women, such as premature ovarian insufficiency (POI). Although many infertility issues have been addressed and resolved through ART, certain limitations persist, and new challenges continue to emerge due to evolving social and biological factors.

Several studies have used human and animal models to address these limitations. Rodents are the most commonly used reproductive models because of their advantages, including shorter gestation periods, relatively large litter sizes, lower costs, and ease of access during breeding. However, despite these benefits, significant anatomical and reproductive physiological differences between rodents and humans limit the applicability of these findings for scientific interpretation and therapeutic advancements. These limitations have driven the need for models resembling human reproductive and endocrine systems. Non-human primates (NHPs) outperform rodent models due to several key human physiological and reproductive similarities. NHPs have been adopted for primary and therapeutic advancements in various fields, including tissue engineering applications. In the realm of reproduction, tissue engineering intersects with what is commonly referred to as reproductive engineering. This discipline encompasses many fields, including embryo and stem cell research, reproductive tract engineering, ARTs, and the generation of transgenic marmosets through genetic manipulation.

Here, we review some studies on reproductive engineering using nonhuman primates (NHPs), focusing on marmosets.

## 2 NHP as a reproductive study model

NHPs serve as crucial reproductive research models due to their genetic, anatomical, and physiological similarities to humans. Their reproductive systems, endocrine cycles, and gestational processes are more similar to those of humans than traditional rodent models, making them invaluable for studies in which direct human experimentation is not feasible.

NHPs have facilitated advanced investigations into fertility preservation, embryology, ART, and the effects of genetic and environmental factors on reproduction. Species such as the common marmoset (*Callithrix jacchus*) are particularly favored for reproductive studies because of their short gestation periods and suitability for genetic manipulation. These characteristics make NHPs essential for bridging the gap between preclinical studies in rodents and human clinical applications, advancing fundamental science and therapeutic innovations in reproductive health (Yun et al., 2016) (Table 1).

**TABLE 1:** Reproductive characteristics of commonly studied monkeys.

Aspect	Rhesus macaque, cynomolgus macaque	Common marmoset
Reproductive System Similarity	Closer to human	More distantly related to humans, but useful for certain reproductive studies
Gestation Period	Longer, around 5–6 months (e.g., macaques, baboons)	Shorter gestation period, around 150 days
Offspring	One offspring per birth	Frequently give birth to twins or triplets
Reproductive Cycle	Ovarian cycle is similar to humans, with menstrual cycles	Most species have estrous cycles
Reproductive Senescence	Female reproductive aging is well-documented, particularly in macaques	Reproductive aging is less understood but may be similar
Model for Human Reproduction	Excellent model for studying human-like reproductive processes (e.g., fertility, menopause, IVF)	Useful for studying reproductive behaviors, genetic studies, and some aspects of early development
Use in ART (Assisted Reproductive Technology)	Frequently used in ART research, including IVF, embryo transfer, and gamete cryopreservation	Used in ART research, particularly in species with more cooperative breeding behaviors (e.g., marmosets)
Ethical Considerations	Higher ethical scrutiny due to their closer genetic relation to humans and more complex social structures	Ethical considerations also present, especially for smaller species like tamarins, due to social dynamics and cooperative breeding

Rhesus monkeys and cynomolgus monkeys have menstrual cycles remarkably similar to humans. This is crucial for studying female reproductive biology, hormonal regulation, and disorders related to menstruation, ovulation, and pregnancy. The hormonal signaling in NHPs, such as estrogen, progesterone, and luteinizing hormone (LH), aligns more closely with that of humans than rodents, which makes NHPs ideal for studying hormonal regulation in reproductive technologies. NHPs have a more comparable reproductive lifespan and fertility pattern to humans, unlike rodents, which have much shorter cycles and faster aging processes. This makes NHPs particularly useful for studying age-related fertility decline, menopause, and related treatments. The gestation period in NHPs is also closer to humans, allowing for more accurate modeling of pregnancy, fetal development, and complications such as preeclampsia or ectopic pregnancies. Conversely, rodents have much shorter gestation periods and are less ideal for long-term gestational studies. These characteristics of NHP considered them more susceptible to certain human-specific reproductive disorders, such as endometriosis and ovarian cancer, making them superior models for understanding these diseases and testing potential treatments. The molecular and genetic profiles of NHPs are far more similar to those of humans than rodents. This allows for studying disease mechanisms at the molecular level in ways that rodent models cannot fully replicate.

NHPs provide a unique and invaluable platform for reproductive engineering. Their use in research enhances the accuracy of studies and improves their successful translation into human therapies, making them indispensable in this field.

## 3 Types of NHPs

NHPs used in reproductive research and other biomedical fields are categorized into two primary groups: New World Monkeys and Old World Monkeys, with a few excellent ape species occasionally included (Mattison and Vaughan, 2017). Each type offers unique advantages and is selected based on the specific requirements of the study. New World monkeys are preferred because of their ease of handling, shorter reproductive cycles, and emerging genetic tools (e.g., marmosets). Old World monkeys are favored because of their physiological and anatomical similarities to humans, making them highly relevant for advanced reproductive studies. Great Apes are limited in use due to ethical constraints but are occasionally studied for insights into human-like reproductive processes. Each type is selected based on the study’s specific goals, balancing relevance to human biology, ethical considerations, and practical factors such as cost and maintenance. The representative NHP types have been discussed ahead.

### 3.1 New World Monkeys

New World monkeys, or platyrrhines, are diverse primates native to Central and South America. They represent one of the two major branches of simian primates, along with the Old World monkeys and apes (catarrhines). “Platyrrhine” means “flat-nosed,” referring to their characteristic broad, outward-facing nostrils. These monkeys are known for their arboreal lifestyles, adaptations to forest environments, and diverse social behaviors.

#### 3.1.1 Common marmoset (*Callithrix jacchus*)

Marmosets are compact primates, weighing approximately 300–500 g. Their small size makes them easier to house, transport, and handle in laboratory settings than larger primates like macaques. Their short reproductive cycles (∼4.5 months) enable faster breeding and the generation of multiple offspring in a shorter time frame, accelerating genetic and developmental studies (Tardif et al., 2003). Marmosets’ hormonal cycles and reproductive physiology are similar to those of humans, making them an excellent model for studying human reproductive biology, fertility, and ART. They regularly produce twins or even triplets, allowing researchers to study sibling interactions and development (Riesche et al., 2018). Their high fertility and genetic manipulability make them an ideal candidate for transgenic studies (Abe et al., 2021). Marmosets are also used to study the hypothalamic-pituitary-adrenal axis and hormonal responses due to their endocrine system similarities with humans (Pryce et al., 2002). Additionally, they require less space, food, and specialized care than larger primates, such as macaques and baboons, making them cost-effective for long-term studies. Although they are not as closely related to humans as Old World monkeys, they are sufficiently similar for many types of biomedical research.

These features make New World monkeys invaluable in various scientific disciplines, bridging the gap between rodent models and larger primates. Their versatility and low resource demands ensure their continued importance as critical model organisms in modern research.

### 3.2 Old World monkeys (catarrhines)

Old World monkeys (Catarrhines) belong to the superfamily Cercopithecoidea and are primarily distributed across Africa and Asia. They form one of the two major branches of simian primates alongside New World monkeys (Platyrrhines). “Old World” refers to their geographic origins in the Eastern Hemisphere.

#### 3.2.1 Rhesus macaque (*Macaca mulatta*)

Rhesus monkeys share approximately 93% of their DNA with humans, making them valuable models for studying human biology (Wolfe, 1983). Their menstrual cycles (28–32 days) and reproductive physiology, including hormonal profiles, are highly analogous to those of humans (Weinbauer et al., 2008). They have been extensively studied in the contexts of IVF, intracytoplasmic sperm injection (ICSI), embryo transfer, menopause, ovarian reserve, and aging-related infertility (Wei et al., 2021). Additionally, they are used to examine early embryonic development, placental function, and maternal-fetal interactions (Niu et al., 2019).

Decades of research have established robust datasets on their reproductive anatomy, physiology, and developmental biology. Standardized protocols and widespread familiarity among researchers have facilitated consistent and reproducible results.

#### 3.2.2 Cynomolgus macaque (*Macaca fascicularis*)

Cynomolgus monkeys have reproductive anatomy and physiology comparable to those of rhesus macaques. They are often more accessible and cost-effective, particularly in regions where they are commonly bred. They are widely used to evaluate reproductive toxicity and the safety of hormonal and contraceptive therapies (Jarvis et al., 2010; Li X. T. et al., 2023). Treatments targeting reproductive hormones, such as estrogen and progesterone, have been tested and validated in these monkeys (Williams et al., 2001). They are also easier to handle and maintain than are rhesus macaques.

#### 3.2.3 Baboon (Papio spp.)

The larger body size of baboons allows for surgical intervention and in-depth studies of reproductive organs. Their reproductive systems closely resemble those of humans, making them particularly valuable for pregnancy-related studies (Bauer, 2015). They investigate the pathophysiology, progression, and treatment of endometriosis, fetal growth, placental biology, and maternal-fetal nutrient exchange (Nyachieo et al., 2007). Researchers also examine conditions such as preeclampsia, gestational diabetes, and spontaneous abortion. Their size and reproductive biology make them ideal for studying conditions challenging to replicate in smaller species or rodents (D'Hooghe et al., 2004). years of research have yielded detailed knowledge regarding baboons’ reproductive cycles and hormone profiles.

## 4 Applications of NHPs in reproductive engineering

NHPs play a crucial role in reproductive research by bridging the gap between rodent models and human clinical studies. Their close genetic and physiological similarities to humans make them indispensable for advancing reproductive science. Below are some notable applications of NHPs in this field.

### 4.1 Assisted reproductive technology (ART)

NHPs, particularly rhesus macaques and marmosets (Buse et al., 2008), are critical in advancing ART, including IVF (Arthur Chang and Chan, 2011). Optimized protocols for these species, including oocyte retrieval, embryo culture, and transfer, are vital for their conservation and study and serve as highly translational models for human fertility treatments (Bavister et al., 1984; Ramsey and Hanna, 2019). These studies provide insights into human reproduction due to the close physiological and genetic similarities between NHPs and humans (Table 2).

**TABLE 2:** Assisted reproductive technology (ART)-related studies.

Monkey species	Age	Title	Research center	References
Rhesus Macaque	N/A	Birth of rhesus monkey infant after *in vitro* fertilization and nonsurgical embryo transfer	Wisconsin Regional Primate Research Center, University of Wisconsin-Madison, United States	Bavister et al. (1984)
Cynomolgus Macaque	N/A	Efficient reproduction of cynomolgus monkey using pronuclear embryo transfer technique	State Key Laboratory of Primate Biomedical Research, Institute of Primate Translational Medicine, Kunming University of Science and Technology, China	Sun et al. (2008)
Rhesus Macaque	6–15 years	Assisted reproductive technology in nonhuman primates	N/A	Arthur Chang and Chan (2011)
Rhesus Macaque	N/A	Generation of chimeric rhesus monkeys	Oregon National Primate Research Center (ONPRC), United States	Tachibana et al. (2012)
Rhesus Macaque	6–12 years	Gonadotropin ratio affects the *in vitro* growth of rhesus ovarian preantral follicles	Dept of OBGY, Seoul National University Hospital, South Korea	Kim et al. (2016)
Cynomolgus Macaque	4.5–9 years	Efficient production of cynomolgus monkeys with a toolbox of enhanced assisted reproductive technologies	College of Veterinary Medicine, South China Agricultural University, China	Ma et al. (2016)
Common Marmoset	3.1–7.5 years	Quality of common marmoset *(Callithrix jacchus)* oocytes collected after ovarian stimulation	Hiroshima University, Japan	Kanda et al. (2018)
Cynomolgus Macaque	7–10 years	*In vitro* culture of cynomolgus monkey embryos beyond early gastrulation	Chinese Academy of Sciences, China	Ma et al. (2019)
Cynomolgus Macaque	6–12 years	*In vitro* culture of embryos from the cynomolgus macaque *(Macaca fascicularis)*	N/A	Curnow and Hayes (2019)
Rhesus Macaque	6–12 years	*In vitro* culture of rhesus macaque *(Macaca mulatta)* embryos	N/A	Ramsey and Hanna (2019)
Cynomolgus Macaque	N/A	Dissecting primate early post-implantation development using long-term *in vitro* embryo culture	State Key Laboratory of Primate Biomedical Research, Institute of Primate Translational Medicine, Kunming University of Science and Technology, China	Niu et al. (2019)
Rhesus Macaque	7–8 years	Metabolomics analysis of follicular fluid coupled with oocyte aspiration reveals the importance of glucocorticoids in primate periovulatory follicle competency	Oregon National Primate Research Center (ONPRC), United States	Ravisankar et al. (2021)
Various	N/A	Ultrasonography of the neotropical primate female reproductive system	N/A	Domingues et al. (2023)

N/A: not available.

A significant contribution of NHP research lies in overcoming challenges associated with male infertility. Precision techniques such as intracytoplasmic sperm injection (ICSI), which involves the direct injection of a single sperm into an oocyte, have been refined using NHP models (Mitalipov et al., 2001; Nusser et al., 2001). These advancements have improved success rates in cases of severe male infertility, including azoospermia (absence of sperm in semen) or poor sperm motility. In an *in vitro* follicular maturation setting, optimal culture conditions and ratios of gonadotropin treatment were reported in a rhesus monkey model (Kim et al., 2016). NHP research also supports the development of pre-implantation genetic testing and optimization of cryopreservation methods to ensure high-quality gametes and embryos for IVF procedures (Sun et al., 2008; Ma et al., 2016; Motohashi and Ishibashi, 2016; Curnow and Hayes, 2019; Ma et al., 2019). These innovations directly inform clinical practice, benefiting individuals and couples facing infertility challenges.

### 4.2 Fertility preservation

Studies on cryopreservation techniques in NHPs have significantly advanced fertility preservation strategies for humans, particularly for individuals facing fertility risks from medical treatments such as chemotherapy (Table 3). Freezing and thawing of sperm, oocytes, and embryos in NHPs offer valuable insights into optimizing protocols for preserving gamete and embryo viability after thawing and minimizing damage caused by ice crystal formation or osmotic stress (Morrell and Hodges, 1998; Jahnukainen et al., 2007; Motohashi and Ishibashi, 2016; Fayomi et al., 2019). These refinements enhance the preservation of human fertility, making options such as embryo or oocyte banking more reliable.

**TABLE 3:** Fertility preservation studies.

Monkey species	Age	Title	Fertility preservation option	References
Rhesus Macaque	18, 21 months of age	Effect of cold storage and cryopreservation of immature non-human primate testicular tissue on spermatogonial stem cell potential in xenografts	Standard freezing using Ethylene glycol and DMSO	Jahnukainen et al. (2007)
Common Marmoset	0–10 days	Cryopreservation of ovaries from neonatal marmoset monkeys	Vitrification	Motohashi and Ishibashi (2016)
Rhesus Macaque	5 years	Subcutaneous ovarian tissue transplantation in nonhuman primates: duration of endocrine function and normalcy of subsequent offspring as demonstrated by reproductive competence, oocyte production, and telomere length	Ovarian cortical tissue transplantation	Lee et al. (2017)
Common Marmoset	1.5–4 years	Expression of transcripts in marmoset oocytes retrieved during follicle isolation without gonadotropin induction	*In vitro* maturation of oocyte	Kim et al. (2019)
Rhesus Macaque	Puberty	Autologous grafting of cryopreserved prepubertal rhesus testis produces sperm and offspring	Testis cryopreservation	Fayomi et al. (2019)
Rhesus Macaque, Cynomolgus macaque, Common Marmoset (male)	N/A	Comparative computer-assisted sperm analysis in non-human primates	Sperm kinematic parameter	Schmidt et al. (2021)
Common Marmoset	1–15 years	Production of marmoset eggs and embryos from xenotransplanted ovary tissues	Ovary transplantation	Hirayama et al. (2023)
Various	N/A	Best practices for cryopreserving sperm in nonhuman primates: a systematic review and meta-analysis	Cryopreserving sperm	Sadeghi et al. (2025)

N/A: not available.

Additionally, NHP models have been instrumental in pioneering ovarian tissue cryopreservation and transplantation (Brito et al., 2017). Research using these models has demonstrated the feasibility of grafting freeze-thawed ovarian tissue to restore endocrine function and fertility (Lee et al., 2017). This technique holds particular promise for patients with prepubertal cancer or women unable to undergo conventional fertility preservation methods. Successes in rhesus macaques and other NHPs have laid the groundwork for translating ovarian tissue transplantation into human clinical applications, where it can restore hormone production, menstrual cycles, and the potential for natural conception.

These advancements underscore the critical role of NHP research in developing innovative fertility preservation techniques, offering hope to individuals facing medical conditions that compromise their reproductive health.

### 4.3 Pluripotent stem cells and germline engineering

NHPs have been pivotal in advancing stem cell research, primarily through deriving gametes from pluripotent stem cells (PSCs) (Mishra et al., 2016; Wu et al., 2023). Various embryonic stem cells (ESCs) and induced pluripotent stem cells (iPSCs) lines have been derived from NHPs (Fang et al., 2014; Honda et al., 2017; Kishimoto et al., 2021). By leveraging their close genetic and physiological similarities to humans, NHPs provide critical insights into germline development and serve as a robust model for translating findings into human medicine (Hayashi et al., 2012) (Table 4).

**TABLE 4:** Pluripotent stem cell and germline engineering.

Generation of PSCs and chimeric embryos using NHPs						
Monkey species	Types	Title	Manipulated genes	Manipulation methods	Research center	References
Rhesus Macaque	iPSCs	Generation of naive induced pluripotent stem cells from rhesus monkey fibroblasts	N/A	N/A	Peking University, China	Fang et al. (2014)
Cynomolgus Macaque	ESCs	Discrimination of stem cell status after subjecting cynomolgus monkey pluripotent stem cells to naïve conversion	N/A	N/A	RIKEN BioResource Center, Japan	Honda et al. (2017)
Rhesus Macaque	Blastocyst	Single-cell RNA sequencing reveals the existence of naive and primed pluripotency in pre-implantation rhesus monkey embryos	N/A	N/A	State Key Laboratory of Primate Biomedical Research, Institute of Primate Translational Medicine, Kunming University of Science and Technology, China	Liu et al. (2018)
Common Marmoset	ESCs	Primed to naive-like conversion of the common marmoset embryonic stem cells	N/A	N/A	Central Institute for Experimental Animals, Japan	Shiozawa et al. (2020)
Cynomolgus Macaque	Human-monkey chimeric embryos	Chimeric contribution of human extended pluripotent stem cells to monkey embryos *ex vivo*	N/A	Blastocyst microinjection	State Key Laboratory of Primate Biomedical Research, Institute of Primate Translational Medicine, Kunming University of Science and Technology, China	Tan et al. (2021)
Common Marmoset	ESCs	Establishment of novel common marmoset embryonic stem cell lines under various conditions	N/A	N/A	Central Institute for Experimental Animals, Japan	Kishimoto et al. (2021)
Rhesus Macaque	Primate cross species embryos	Chimpanzee and pig-tailed macaque iPSCs: improved culture and generation of primate cross-species embryos	BCLs	Microinjection	California National Primate Research Center (CNPRC), United States	Roodgar et al. (2022)
Cynomolgus Macaque, Rhesus Macaque (male and female)	5–10 years	Long-term *in vivo* chimeric cells tracking in non-human primate	GFP	Intravenous injection	State Key Laboratory of Primate Biomedical Research, Institute of Primate Translational Medicine, Kunming University of Science and Technology, China	Wu et al. (2024)

Germ cell generation				
Monkey species	Age	Title	Research center	References
Common Marmoset	newborn, 8-week, adult marmoset monkeys	Comparative marker analysis after isolation and culture of testicular cells from the immature marmoset	German Primate Center, Göttingen, Germany	Albert et al. (2012)
Cynomolgus Macaque	N/A	The germ cell fate of cynomolgus monkeys is specified in the nascent amnion	Kyoto University, Shiga University of Medical Science, Japan	Sasaki et al. (2016)
Common Marmoset	N/A	Efficient generation of marmoset primordial germ cell-like cells using induced pluripotent stem cells	Southwest National Primate Research Center (SNPRC), Texas Biomedical Research Institute, United States	Seita et al. (2023)

*ESCs: embryonic stem cells, iPSCs: induced pluripotent stem cells, N/A: not available.

Researchers have successfully reprogrammed iPSCs and ESCs into germline-like cells in NHPs, enabling detailed investigations into mechanisms regulating spermatogenesis and oogenesis (Makar and Sasaki, 2020). These studies have identified key signaling pathways and genetic factors required for proper germ cell differentiation (Sasaki et al., 2016; Liu et al., 2018). They also offer a controlled environment for exploring interactions between germline cells and their surrounding somatic niches, which are essential for gamete maturation (Seita et al., 2023).

The ability to derive functional gametes from stem cells represents a groundbreaking advancement in addressing infertility. This research offers hope for future therapeutic interventions for individuals with conditions such as gonadal dysgenesis, cancer treatment-induced gonadal damage, or genetic mutations impairing germ cell production (Tan et al., 2021; Roodgar et al., 2022). By generating gametes *in vitro*, researchers aim to restore fertility in cases where native germ cells are absent or non-functional.

NHP models are also instrumental in ensuring the safety and efficacy of these approaches, particularly in validating whether stem cell-derived gametes can undergo regular fertilization and produce healthy offspring (Rodriguez-Polo and Behr, 2022). These findings are critical for clinical applications, ensuring these techniques are robust and applicable to human reproductive medicine.

### 4.4 Female reproductive tract (FRT) engineering

Tissue engineering of NHPs has opened new frontiers in reproductive medicine by providing a platform for the development of artificial uterine and ovarian tissues. These advances could replace damaged reproductive organs and enhance the success of ART (Johannesson et al., 2012) (Table 5).

**TABLE 5:** Female reproductive tract (FRT) engineering.

Monkey species	Age	Model disease	Title	Research center	References
Baboon	8–13 years	Premature ovarian insufficiency (POI)	A modified baboon model for endometriosis	University of Illinois at Chicago, United States Institute for Primate Research in Nairobi, Kenya	Fazleabas et al. (2002)
Rhesus Macaque	8–9 years	N/A	Encapsulated three-dimensional culture supports development of nonhuman primate secondary follicles	Oregon National Primate Research Center (ONPRC), United States	Xu et al. (2009)
N/A	N/A	N/A	*In vitro* modeling of the physiological and diseased female reproductive system	Department of Bioengineering, Imperial College London, London, United Kingdom	Stejskalova et al. (2021)
Old world and new world monkeys	N/A	Spontaneous urogenital lesions	Urogenital lesions in nonhuman primates at 2 national primate research centers	Emory University, United States	Kirejczyk et al. (2021)
Cynomolgus Macaque	8–13 years	Premature ovarian insufficiency (POI)	Autologous transplantation of thecal stem cells restores ovarian function in nonhuman primates	Center for Stem Cell Biology and Tissue Engineering, Key Laboratory for Stem Cells and Tissue Engineering, Sun Yat-sen University, China	Chen et al. (2021)
Rhesus Macaque	10 years	Pelvic organ prolapse (POP)	Mesenchymal stem cell-based bioengineered constructs enhance vaginal repair in ovariectomized rhesus monkeys	Chinese Academy of Sciences, China	Ma et al. (2021)
Common Marmoset	N/A	Diminished ovarian reserve (DOR)	Synergistic promoting effects of X-linked inhibitor of apoptosis protein and matrix on the *in vitro* follicular maturation of marmoset follicles	Dept of OBGY, Seoul National University Hospital, South Korea	Kim et al. (2022)
Baboons *(Papio hamadryas)*	N/A	Uterine factor infertility	Toward human uterus tissue engineering: Uterine decellularization in a non-human primate species	Laboratory for Transplantation and Regenerative Medicine, Sahlgrenska Academy University of Gothenburg Gothenburg Sweden	De Miguel-Gomez et al. (2024)

N/A: not available.

#### 4.4.1 Artificial ovarian tissue

Researchers have focused on engineering functional ovarian tissues that mimic the ovaries’ natural hormonal and gametogenic functions. Artificial ovarian constructs have been developed using NHP models by incorporating ovarian cells or follicles into biocompatible scaffolds (Chen et al., 2021). These scaffolds provide structural support while facilitating cell survival, vascularization, and follicular maturation (Kim et al., 2022).

This technology could benefit individuals who have lost ovarian function owing to conditions such as POI, cancer treatment, or age-related decline. By restoring endocrine activity, these artificial tissues can support natural hormonal cycles, and the maturation of enclosed follicles may offer an alternative source of oocytes for IVF.

#### 4.4.2 Artificial uterine tissue

Studies using NHPs have explored the engineering of artificial uterine tissues. Researchers have attempted to create a functional uterine environment that supports embryo implantation and development by combining endometrial cells with bioengineered scaffolds. These studies are critical for individuals with uterine abnormalities, such as Asherman syndrome or congenital uterine malformations, which impair fertility (Kirejczyk et al., 2021). Artificial uterine constructs could provide an option for women who are unable to conceive because of uterine damage, offering hope for biological parenthood without relying on surrogacy.

#### 4.4.3 NHPs as models for clinical translation

Owing to their physiological similarities to humans, NHPs are an ideal model for developing and testing tissue engineering technologies (Daadi et al., 2014). They provide critical data on the biocompatibility, functionality, and long-term viability of artificial tissues in the reproductive context (Stejskalova et al., 2021). Research on NHPs has ensured that these engineered constructs can be integrated with native tissues, support normal reproductive processes, and maintain safety before clinical application in humans (Ma et al., 2021).

### 4.5 Reproductive endocrinology, infertility, and pregnancy

NHPs have advanced our understanding of hormonal reproductive regulation. They offer insight into diagnosing and treating human reproductive disorders (Fazleabas, 2006; Kyama et al., 2007; Taylor et al., 2017). Their close resemblance to human reproductive physiology makes them invaluable for studying complex hormonal interactions during the menstrual cycle, pregnancy, and related conditions (Braundmeier et al., 2012) (Table 6).

**TABLE 6:** Studies of reproductive endocrinology, infertility, and pregnancy.

Reproductive endocrinology and infertility							
Monkey species	Age	Model disease	Title	Manipulated genes	Manipulation methods	Research center	References
Rhesus Macaque	5–12 months	Polycystic ovary syndrome	Fetal, infant, adolescent and adult phenotypes of polycystic ovary syndrome in prenatally androgenized female rhesus monkeys	N/A	N/A	Wisconsin National Primate Research Center, University of Wisconsin, United States	Abbott et al. (2009)
Olive Baboons	Reproductive age	Endometriosis	Induction of endometriosis alters the peripheral and endometrial regulatory T cell population in the non-human primate	N/A	N/A	University of Illinois, United States	Braundmeier et al. (2012)
Rhesus Macaque	7–8 years	N/A	Metabolomics analysis of follicular fluid coupled with oocyte aspiration reveals the importance of glucocorticoids in primate periovulatory follicle competency	NR3C1	Morpholino antisense oligonucleotide (MAO)	Oregon National Primate Research Center (ONPRC), United States	Ravisankar et al. (2021)

Pregnancy					
Monkey species	Modeling	Title	Research center	References
Rhesus Macaque	Abdominal aortas	Increased depth of trophoblast invasion after chronic constriction of the lower aorta in rhesus monkeys	University of California, United States	Zhou et al. (1993)	
Common Marmoset	Postnatal growth	Relations among birth condition, maternal condition, and postnatal growth in captive common marmoset monkeys	Southwest National Primate Research Center (SNPRC), Texas Biomedical Research Institute, United States	Tardif and Bales (2004)	
Cynomolgus Macaque (female)	Human development	Dissecting primate early post-implantation development using long-term *in vitro* embryo culture	State Key Laboratory of Primate Biomedical Research, Institute of Primate Translational Medicine, Kunming University of Science and Technology, China	Niu et al. (2019)	
Rhesus Macaque (pregnant)	3.5 years	Preliminary evidence of increased striatal dopamine in a nonhuman primate model of maternal immune activation	Department of Psychiatry and Behavioral Sciences, University of California, United States	Bauman et al. (2019)	
Common Marmoset (female)	N/A	Single cell transcriptome analysis of human, marmoset and mouse embryos reveals common and divergent features of preimplantation development	Xieerxin Biology Resource with accreditation of Laboratory Animal Care accredited facility in Beijing, China	Wang et al. (2020b)	
Rhesus Macaque	N/A	Micro-anatomic alterations of the placenta in a non-human primate model of gestational protein-restriction	The University of Texas Health Science at Houston, United States	Sargent et al. (2020)	
Rhesus Macaque	Teratogenesis	Non-human primate models to investigate mechanisms of infection-associated fetal and pediatric injury, teratogenesis and stillbirth	Primate Research Center, Peking University, China	Li et al. (2021)	

N/A: not available.

PCOS studies in NHPs have examined the effects of excess androgens on follicular development and insulin resistance, thereby providing a preclinical model for testing treatments (Abbott et al., 1998; Abbott et al., 2017). Similarly, NHP studies on menstrual physiology and endometrial lesions have advanced our understanding of the inflammatory and hormonal mechanisms underlying endometriosis (Barrier et al., 2007; Wang et al., 2009).

In particular, marmosets have contributed significantly to understanding hormone signaling between the mother and fetus (Barnett et al., 2006). These studies have revealed how maternal hormones, such as progesterone and cortisol, influence fetal development, placental function, and labor timing (Einspanier et al., 1997; Rutherford et al., 2014). Disruptions in these signaling pathways are implicated in pregnancy complications such as preeclampsia, preterm birth, and intrauterine growth restriction (Wedi et al., 2011).

For instance, research on maternal cortisol regulation in marmosets has highlighted its role in programming fetal stress responses and metabolism, which may have long-term implications for offspring health. These studies provide a foundation for developing interventions to mitigate pregnancy-related complications in humans.

### 4.6 Developmental studies

Studies of early embryonic development in NHPs have provided crucial data on implantation and developmental milestones, offering insights into miscarriage and congenital disorders (Table 7). Research on NHPs has revealed the molecular and cellular processes underlying embryonic attachment to the uterine lining and trophoblast invasion (Zhou et al., 1993), which are crucial for successful implantation (Tardif and Bales, 2004; Ochoa-Bernal and Fazleabas, 2020). These findings help clarify the causes of implantation failure, which is a significant factor in infertility and early pregnancy loss. NHP models allow a detailed examination of embryonic genome activation, cell differentiation, and the formation of critical structures, such as the blastocyst and germ layers (Bergmann et al., 2022; Zhai et al., 2022; Gong et al., 2023; Li X. T. et al., 2023). Insights from these studies are essential to understand how disruptions during early development contribute to miscarriages or congenital disorders.

**TABLE 7:** Developmental studies in NHPs.

Monkey species	Age	Title	Research center	References
Rhesus Macaque	N/A	Differentiation of primate primordial germ cell-like cells following transplantation into the adult gonadal niche	Oregon National Primate Research Center (ONPRC), United States	Sosa et al. (2018)
Rhesus Macaque	8–14 years	Acetylcholine and necroptosis are players in follicular development in primates	Oregon National Primate Research Center (ONPRC), United States	Du et al. (2018)
Rhesus Macaque (male)	2 years	*In vitro* differentiation of rhesus macaque bone marrow- and adipose tissue-derived MSCs into hepatocyte-like cells	State Key Laboratory of Primate Biomedical Research, Institute of Primate Translational Medicine, Kunming University of Science and Technology, China	Wang et al. (2020a)
Cynomolgus Macaques	4–10 years	Granulosa cell proliferation is inhibited by PGE2 in the primate ovulatory follicle	Department of Physiological Sciences, Eastern Virginia Medical School, United States	Lundberg et al. (2020)
Cynomolgus Macaque	5–8 years	Chimeric contribution of human extended pluripotent stem cells to monkey embryos *ex vivo*	State Key Laboratory of Primate Biomedical Research (LPBR), United States	Tan et al. (2021)
Cynomolgus Macaque	6–8 years	Primate gastrulation and early organogenesis at single-cell resolution	UT Southwestern Medical Center, United States	Zhai et al. (2022)
Cynomolgus Macaque	5–12 years	Cynomolgus monkey embryo model captures gastrulation and early pregnancy	CAS Center for Excellence in Brain Science and Intelligence Technology, Chinese Academy of Sciences, China	Li et al. (2023a)
Cynomolgus Macaque	7–10 years	Neurulation of the cynomolgus monkey embryo achieved from 3D blastocyst culture	Xieerxin Biology Resource with the accreditation of the laboratory animal care facility in Beijing, China	Zhai et al. (2023)
Cynomolgus Macaque	5–12 years	Ex utero monkey embryogenesis from blastocyst to early organogenesis	State Key Laboratory of Primate Biomedical Research (LPBR), United States	Gong et al. (2023)
Rhesus Macaque	6–14 years	Dynamic changes in gene expression of growing nonhuman primate antral follicles	Department of Animal Science, Michigan State University, United States	VandeVoort et al. (2024)

N/A: not available.

### 4.7 Reproductive aging

NHP models, such as macaques, have been used to study ovarian aging, menopause, and associated health issues. These models help researchers explore potential interventions to extend reproductive lifespan or mitigate age-related infertility (Hernandez-Lopez et al., 2012). Studies on rhesus macaques have helped to identify biomarkers of ovarian reserves, aiding fertility assessments in humans (Alberts et al., 2013; Lee et al., 2021) (Table 8).

**TABLE 8:** Studies of reproductive aging.

Monkey species	Age	Title	Research center	References
Rhesus Macaque	10–25 years	Neuroendocrine changes in the aging reproductive axis of female rhesus macaques *(Macaca mulatta)*	Oregon National Primate Research Center (ONPRC), United States	Downs and Urbanski (2006)
Rhesus Macaque	4–27 years	Heterogeneity of reproductive aging in free-ranging female rhesus macaques	Miami Dade College and University of Miami, United States	Johnson and Kapsalis (2008)
Cynomolgus Macaque	4.5–12 years	Experimental induction of reduced ovarian reserve in a nonhuman primate model *(Macaca fascicularis)*	Wake Forest University Primate Center, United States	Appt et al. (2010)
Geoffroy’s spider monkey (male)	13–27 years	Aging-related reproductive decline in the male spider monkey *(Ateles geoffroyi)*	Instituto Nacional de Psiquiatría Ramón de la Fuente Muñiz in Mexico City (INPRFM), Mexico	Hernandez-Lopez et al. (2012)
Rhesus Macaque	4–19 years	Age-specific gene expression profiles of rhesus monkey ovaries detected by microarray analysis	South China Agricultural University, China	Wei et al. (2015)
Cynomolgus Macaque (female)	5–20 years	Single-cell transcriptomic atlas of primate ovarian aging	Chinese Academy of Sciences, China	Wang et al. (2020b)
N/A	4–20 years	Single-cell profiling of mouse and primate ovaries identifies high levels of EGFR for stromal cells in ovarian aging	Department of Gynecology and Obstetrics, Huazhong University of Science and Technology, China	Wei et al. (2023)
Cynomolgus Macaque	4–19 years	Aging hallmarks of the primate ovary revealed by spatiotemporal transcriptomics	National Clinical Research Center for Geriatric Disorders, Xuanwu Hospital Capital Medical University, China	Lu et al. (2024)
Cynomolgus Macaque	3–23 years	Stem cell transplantation extends the reproductive life span of naturally aging cynomolgus monkeys	Chinese Academy of Sciences, China	Yan et al. (2024)
Rhesus Macaque	22–28 years	Cellular and molecular mechanisms of highly active mesenchymal stem cells in the treatment of senescence of rhesus monkey ovary	Kunming Medical University, China	Wang et al. (2024)

N/A: not available.

Macaques exhibit reproductive aging patterns similar to humans, including a decline in ovarian reserve, irregular menstrual cycles, and eventual menopause. Research using these models has provided critical insights into mechanisms underlying ovarian aging and menopause (Brenner et al., 2004). Studies in rhesus macaques have highlighted the decline in follicular quantity and quality with age, along with changes in hormone levels (e.g., estradiol, progesterone, and FSH) that mirror human menopausal transitions (Downs and Urbanski, 2006; Johnson and Kapsalis, 2008). NHP models have also been used to explore the systemic effects of ovarian aging, such as increased risks of osteoporosis, cardiovascular disease, and cognitive decline (Garber et al., 2020). These parallels make them ideal models for studying postmenopausal health.

### 4.8 Transcriptomic analyses of the NHP model

Transcriptomic analyses of NHP models have revolutionized our understanding of gene expression dynamics in reproduction, development, aging, and disease (Wang S. et al., 2020; Bergmann et al., 2022) (Table 9). These studies have provided insights into the molecular mechanisms underlying normal physiological and pathological conditions by analyzing a full range of RNA transcripts (Han et al., 2022; Tu et al., 2022).

**TABLE 9:** Transcriptomic analyses of the NHP model.

Monkey species	Age	Title	Genes on focus	Research center	References
Rhesus Macaque (female)	5–12 years	Dynamics of the transcriptome in the primate ovulatory follicle	*HAS-2, TNFAIP6*	Oregon National Primate Research Center (ONPRC), United States	Xu et al. (2011)
Gorilla	N/A	The non-human primate reference transcriptome resource (NHPRTR) for comparative functional genomics	*N/A*	University of Washington, United States	Pipes et al. (2013)
Cynomolgus Macaque	4–5 years; 18–20 years	Single cell transcriptome analysis of human, marmoset and mouse embryos reveals common and divergent features of preimplantation development	*OTX2*	Wellcome Trust – Medical Research Council Stem Cell Institute, University of Cambridge, United Kingdom	Boroviak et al. (2018)
Cynomolgus Macaque	5–20 years	Single-cell transcriptomic atlas of primate ovarian aging	*IDH1, NDUFB10*	Chinese Academy of Sciences, China	Wang et al. (2020b)
Cynomolgus Macaque (female)	embryonic days 84 and 116	Single-cell RNA sequencing reveals regulation of fetal ovary development in the monkey (*Macaca fascicularis*)	*ZGLP1*	Chinese Academy of Sciences, China	Zhao et al. (2020)
Cynomolgus Macaque	random	A reference single-cell regulomic and transcriptomic map of cynomolgus monkeys	*S4A48, CD72* *FEV*	Nanjing University, China	Qu et al. (2022)
Common Marmoset (female)	8–11 years	Spatial profiling of early primate gastrulation *in utero*	*NODAL, WNT*	University of Cambridge, United Kingdom	Bergmann et al. (2022)
Cynomolgus Macaque	6 years	Cell transcriptomic atlas of the non-human primate *Macaca fascicularis*	*ACE2, TMPRSS2*	BGI-Shenzhen, Shenzhen, China. 2 BGI-Beijing, Beijing, China	Han et al. (2022)
Cynomolgus Macaque	16–18 years	Deciphering the dynamics of the ovarian reserve in cynomolgus monkey through a quantitative morphometric study	*PTEN, SOHLH2*	Chinese Academy of Sciences, China	Tu et al. (2022)
Common Marmoset (female)	1.5–9 years	Transcriptomic profiling of reproductive age marmoset monkey ovaries	*N/A*	Dept of OBGY, Seoul National University Hospital, South Korea	Kim et al. (2024)

N/A: not available.

Transcriptomic analyses of NHPs have examined age-related changes in various tissues, including ovaries, testes, brain, and muscles (Xu et al., 2011; Zhao et al., 2020; Kim et al., 2024). Comparative transcriptomic analyses between NHPs and humans can help identify evolutionary changes in gene expression that may explain species-specific traits, including cognitive abilities, immune responses, and reproductive strategies.

### 4.9 Generation of transgenic NHPs

NHP embryos have been used to refine CRISPR-Cas9 genome-editing techniques and test the precision and safety of this technology in correcting genetic mutations (Nusser et al., 2001; Liu et al., 2014; Chen et al., 2015; Sato et al., 2016; Kumita et al., 2019; Abe et al., 2021; Ryu et al., 2022; Seita et al., 2023). Generating genetically modified marmosets enables researchers to study gene functions related to reproduction and developmental biology, such as genes controlling spermatogenesis or the ovarian reserve (Sasaki et al., 2009; Yoshimatsu et al., 2019; Tomioka et al., 2020; Drummer et al., 2021).

These studies are valuable in addressing infertility associated with hereditary diseases or genetic mutations. Moreover, research involving NHPs ensures these methods are safe and effective before transitioning to human clinical applications (Table 10).

**TABLE 10:** Generation of transgenic NHPs.

Transgenic NHP model production					
Monkey species	Model disease	Title	Manipulated genes	Manipulation methods	References
Common Marmoset	N/A	Generation of transgenic non-human primates with germline transmission	*EGFP*	Microinjection	Sasaki et al. (2009)
Rhesus Macaque	ICM	Generation of chimeric rhesus monkeys	*EGFP*	Microinjection	Tachibana et al. (2012)
Cynomolgus Macaque	N/A	Generation of gene-modified cynomolgus monkey via Cas9/RNA-mediated gene targeting in one-cell embryos	Ppar-g, Rag1	CRISPR/Cas9	Niu et al. (2014)
Rhesus Macaque, Cynomolgus Macaque	X-linked, Rett syndrome (RTT)	TALEN-mediated gene mutagenesis in rhesus and cynomolgus monkey	*MECP2*	TALEN	Liu et al. (2014)
Cynomolgus Macaque	N/A	Generation of cynomolgus monkey chimeric fetuses using embryonic stem cells	*green fluorescent protein (GFP)*	Microinjection	Chen et al. (2015)
Common Marmoset	N/A	Generation of a nonhuman primate model of severe combined immunodeficiency using highly efficient genome editing	*IL2RG*	ZFNs/TALENs	Sato et al. (2016)
Cynomolgus Macaque	N/A	Generation of transgenic cynomolgus monkeys that express green fluorescent protein throughout the whole body	*green fluorescent protein (GFP)*	Lentivirus injection	Seita et al. (2016)
Common Marmoset	N/A	Generation and breeding of EGFP-transgenic marmoset monkeys: cell chimerism and implications for disease modeling	*EGFP*	Lentivirus injection	Drummer et al. (2021)
Common Marmoset	Spinocerebellar Ataxia Type 3	Generation of common marmoset model lines of spinocerebellar ataxia type 3	*SCA3*	N/A	Tomioka et al. (2020)
Rhesus Macaque	Usher syndrome type 1B	CRISPR/Cas9 editing of the myo7a gene in rhesus macaque embryos to generate a primate model of usher syndrome type 1b	*MYO7A*	CRISPR/Cas9	Ryu et al. (2022)
Rhesus Macaque	Huntington’s disease (HD)	Generation of rhesus macaque embryos with expanded cag trinucleotide repeats in the huntingtin gene	*HTT*	CRISPR/Cas9	Ryu et al. (2024)

Methods of producing transgenic NHPs							
Monkey species	Age	Model disease	Title	Manipulated genes	Manipulation methods	Research center	Reference
Common Marmoset	N/A	Target gene KI/KO model	Efficient marmoset genome engineering by autologous embryo transfer and CRISPR/Cas9 technology	*c-kit; shank3*	CRISPR/Cas9	Central Institute for Experimental Animals, Japan	Abe et al. (2021)
Common Marmoset	2–8 years	FMR1mutant model	Efficient marmoset genome engineering by autologous embryo transfer and CRISPR/Cas9 technology	*FMR1*	AET; CRISPR/Cas9	University of Tokyo, Japan	Kumita et al. (2019)
Common Marmoset	2–6 years	N/A	Robust and efficient knock-in in embryonic stem cells and early-stage embryos of the common marmoset using the CRISPR-Cas9 system	*FOXP2, PLP1*	CRISPR/Cas9	RIKEN Institute, Japan	Yoshimatsu et al. (2019)

N/A: not available.

### 4.10 Ethical consideration

Non-human primates (NHPs) use in reproductive engineering, particularly in fields like assisted reproductive technology (ART) and stem cell research, raises several ethical concerns related to animal welfare. These concerns stem from the study’s complexity, the procedures’ invasiveness, and the potential for suffering in NHPs. For the welfare of the NHP, the following points should be considered.

Reproductive engineering techniques often involve highly invasive procedures such as oocyte retrieval, embryo transfer, or hormonal manipulation. To minimize distress and harm, these procedures should be performed with the utmost care and precision, ideally under anesthesia or appropriate analgesia, and only when necessary for the research objectives. Effective anesthesia and analgesia should be administered to minimize pain and discomfort, and the animals should be monitored continuously to detect signs of distress or discomfort. Where possible, researchers should explore non-invasive or minimally invasive techniques to gather data, such as non-invasive imaging, blood or urine analysis, and genomic studies.

Genetic modifications (e.g., CRISPR) for reproductive engineering in NHPs raise additional ethical concerns, especially regarding unintended genetic consequences and the long-term impact on animal welfare. Ethical review is crucial to ensure that such experiments are conducted with appropriate safeguards and that potential risks are minimized.

Ethical considerations regarding using NHPs in reproductive engineering are complex but crucial. They revolve around minimizing harm, ensuring scientific necessity, and providing appropriate animal care throughout their lives. Ethical guidelines, such as those offered by IACUCs and institutional review boards, exist to ensure that NHPs are treated with respect, their welfare is a top priority, and their use is scientifically justified. Adhering to these principles ensures humane treatment and enhances the credibility and legitimacy of the research.

## 5 Discussion

Reproductive tissue engineering in non-human primates (NHPs) is a critical area of research that focuses on developing new methods to restore, replace, or enhance reproductive function using tissue engineering techniques. This research has significant implications for improving treatments for infertility, reproductive disorders, and advancing technologies like assisted reproductive technology (ART) and gene editing. NHPs are particularly valuable in this field because their reproductive systems are highly similar to humans, making them an ideal model for studying complex reproductive processes.

NHPs are critical models in reproductive studies because of their physiological and genetic similarity to humans. Unlike rodent models, NHPs share similar endocrine profiles, reproductive cycles, and placental structures, essential for translating findings into human applications. The advent of transgenic NHPs has further expanded the possibilities for understanding and addressing complex reproductive disorders. These models facilitate testing innovative ART techniques, such as *in vitro* gametogenesis and uterine bioengineering, under conditions that closely mimic human biology.

One of the major goals of reproductive tissue engineering is to regenerate or replace damaged ovarian tissue, which is critical for female fertility. NHPs have been used to test bioengineered ovarian tissues created from stem cells or decellularized ovarian matrices. These engineered tissues can potentially restore fertility in females who have experienced ovarian failure due to aging, chemotherapy, or genetic conditions.

In NHPs, researchers have investigated how bioengineered ovaries support the maturation of oocytes (eggs) and follicle development *in vitro*. This is an essential step toward creating functional ovarian tissue that can support healthy egg production for ART or fertility preservation.

For women suffering from uterine disorders like Asherman’s syndrome or dysfunctional endometrium, tissue engineering can offer solutions by regenerating the uterine lining (endometrium). NHPs are used to study the implantation process of embryos and the role of the endometrial tissue in successful pregnancy. Tissue-engineered endometrial models derived from stem cells could 1 day be used to treat women with damaged or non-functional uterine linings. Tissue-engineered models of the uterus or endometrium are being studied to improve understanding of embryo implantation, a crucial step in pregnancy. By using NHPs, researchers can explore how various factors, such as hormones or immune responses, influence implantation success and pregnancy outcomes.

Depending on the experimental objective, different NHP species have unique advantages in reproductive research. Marmosets, for example, are small-bodied primates with short gestation periods and frequent twin births, making them ideal for genetic studies. Their rapid reproductive cycles allow the generation of transgenic animals to study specific gene functions during reproduction and early embryonic development. Larger primates have anatomical and physiological features, including uterine size and structure, that closely resemble those of humans. They are preferred for studies involving surgical procedures, such as uterine transplantation, and for developing techniques to improve implantation and pregnancy outcomes.

With the emergence of the NHP model, ethical considerations should be discussed to ensure humane treatment and minimize suffering in experimental settings. Many reproductive engineering procedures, such as oocyte retrieval, embryo culture, and genetic manipulation, are invasive. Ethical guidelines emphasize minimizing harm and ensuring proper pain management, as well as following the 3Rs principle (Replacement, Reduction, and Refinement). Clear guidelines for genetic modification and reproductive experimentation using NHPs, particularly the use of artificial embryos, are essential.

CRISPR-based gene editing in NHPs holds promise for treating genetic infertility disorders or diseases like cystic fibrosis, which impact reproductive organs. Research is focused on editing the genes of sperm or eggs before fertilization to correct genetic defects and potentially eliminate hereditary diseases.

Reproductive engineering using NHP models may lead to further advances in various fields. The application of CRISPR-Cas9 and other precise gene editing technologies to create targeted modifications in NHPs can accelerate the development of genetic tools. These technologies can also be used to explore artificial uteri and tissue engineering to study the implantation and gestation processes *in vitro*. Additionally, long-term follow-ups of transgenic NHPs should be conducted to assess their health, fertility, and genetic stability across generations.

## 6 Conclusion

Reproductive tissue engineering in NHPs is a rapidly advancing field with the potential to revolutionize fertility treatments and our understanding of reproductive biology. By using NHPs as models, researchers can develop more effective ART techniques, regenerative therapies, and personalized treatments for reproductive disorders. However, this work must be balanced with careful ethical considerations, particularly regarding animal welfare and the long-term implications of using advanced technologies like gene editing.
